# Tertiary Lymphoid Structures: Autoimmunity Goes Local

**DOI:** 10.3389/fimmu.2018.01952

**Published:** 2018-09-12

**Authors:** Elena Pipi, Saba Nayar, David H. Gardner, Serena Colafrancesco, Charlotte Smith, Francesca Barone

**Affiliations:** ^1^Rheumatology Research Group, Institute of Inflammation and Ageing, University of Birmingham, Birmingham, United Kingdom; ^2^Experimental Medicine Unit, Immuno-Inflammation Therapeutic Area, GSK Medicines Research Centre, Stevenage, United Kingdom; ^3^Reumatologia, University of Rome, Sapienza, Italy

**Keywords:** tertiary lymphoid structures (TLS), autoantibodies, germinal center response, glycosylation, B-cells

## Abstract

Tertiary lymphoid structures (TLS) are frequently observed in target organs of autoimmune diseases. TLS present features of secondary lymphoid organs such as segregated T and B cell zones, presence of follicular dendritic cell networks, high endothelial venules and specialized lymphoid fibroblasts and display the mechanisms to support local adaptive immune responses toward locally displayed antigens. TLS detection in the tissue is often associated with poor prognosis of disease, auto-antibody production and malignancy development. This review focuses on the contribution of TLS toward the persistence of the inflammatory drive, the survival of autoreactive lymphocyte clones and post-translational modifications, responsible for the pathogenicity of locally formed autoantibodies, during autoimmune disease development.

## Introduction

The polyclonal expansion of autoreactive B cells is a cardinal feature of autoimmune conditions. Whether directed against a single antigen or playing part in a poly-specific response, autoreactive B cells support the persistence of the autoimmune process and, in several cases are directly pathogenic.

The development of an autoreactive B cell repertoire during the natural history of the autoimmune condition is regulated by the process of affinity maturation against single or multiple autoantigens that occurs within the inner part of the B cell follicles, classically within secondary lymphoid organs (SLOs) (1). Formation of B cell follicles and germinal centers (GC) has been also described in ectopic or tertiary lymphoid structures (TLS) in a process defined “ectopic lymphoneogenesis.” TLS form at target organs of chronic inflammatory/autoimmune process, localized infections and in the areas surrounding solid tumors (2–11). The prognostic value of these structures is debated. TLS formation in target organs autoimmune disease is classically associated with disease persistence and worst clinical manifestations. In solid tumors TLS have instead been associated with the generation of an anti-tumor response, however in some cases the ability of tumor cells to induce T regulatory cells (Treg) and suppress the host immune response has been described Table 1.

**Table 1:** TLS in different conditions.

**Disease**	**Type**	**Localization**	**Specific antigens identified?**	**Role/prognosis**	**Human studies**	**Mouse studies**
GPA/WG	AID	Lungs	ANCAs	pathogenic	(12, 13)	
Hashimoto's Thyroiditis	AID	Thyroid	Thyroglobulin, Thyroperoxidase	pathogenic	(14, 15)	(16, 17)
MS	AID	CNS	Myelin (in mice)	pathogenic	(18–21)	(22–28)
Myasthenia gravis	AID	Thymus	Acetylcholine receptor	pathogenic	(29, 30)	(31)
Primary biliary cirrhosis,	AID	Liver	No	N/A	(32)
Rheumatoid Arthritis	AID	Synovium	RF, Citrullinated proteins	pathogenic	(33, 34)	(33, 35)
Sjogren's Syndrome	AID	Salivary/Lachrymal glands, Lung	SSA/Ro & SSB/La	pathogenic	(36–38)	(39, 40)
SLE	AID	Kidneys	No	pathogenic	(41)	(42, 43)
Breast cancer	Can	Breast	Tumor associated antigens	favorable	(44–47)	
Colorectal cancer	Can	Colon	No	favorable	(48, 49)	(49)
Lung cancer	Can	Lung	No	favorable	(50, 51)	
Ovarian cancer	Can	Ovarian	No	favorable	(52)	
Melanoma	Can	Skin	No	favorable	(53)	
PCD	Can	Pancreas	No	favorable	(54)	
Prostate cancer	Can	Prostate	No	favorable	(55)	
Atherosclerosis	CID	Arteries	No	protective (in mice)	(56, 57)	(58, 59)
COPD	CID	Lung	No	pathogenic (in mice)	(60–64)	(60, 62, 65)
IBD	CID	Gut	No	pathogenic (in mice)	(66–69)	(70–74)
PSC	CID	Liver	No	N/A	(75)	
Lyme disease	Inf	Joints	No direct evidence	N/A	(76)	
HCV	Inf	Liver	No direct evidence	N/A	(77–80)	
*Heliobacter pylori*	Inf	Gastric wall	No direct evidence	Pathogenic	(81–83)	(84)
*Mycobacterium tuberculosis*	Inf	Lungs	No direct evidence	Protection against pathogen	(85–87)	(85, 86, 88)
Allograft transplants	Tra	Heart, lung, kidney	Allo-antigens	Highly controversial	(89–94)	(95, 96)

*GPA/WG, Granulomatosis polyangiitis/Wegener's granulomatosis; COPD, Chronic Obstructive Pulmonary Disease; IBD, Inflammatory Bowel Disease; PSC, Primary Sclerosing Cholangitis AID, Autoimmune disease; CID, Chronic inflammatory disease; PDC, Pancreatic duct carcinoma; HCV, Hepatitis C virus; Can, Cancer; Tran, Transplantation; Inf, Infection. (Note: Studies on mice are presented only if there is evidence from human studies for the presence of TLSs in these different conditions)*.

Often indicated as “tertiary lymphoid organs,” TLS fail to adhere to the proper definition of organs as they lack a stable structural organization, including a capsule, and are better classified as “tertiary lymphoid structures” or TLS (97). TLS are not present in embryonic life and form in adult life to support local aggregation of lymphocytes at the target organ of disease. Other terms including “ectopic lymphoid structures” (ELS) or “ectopic germinal centers”. The latter, however, should only be used when GC formation is determined histologically within the ectopic lymphoid tissue (97–101). The cross-over between TLS and SLO is the subject of debate and has been reviewed by ourselves and others in recent publications (9, 98).

The term “tertiary lymphoid” tissue in the literature dates back to 1992 and was introduced by Louis Picker and Eugene Butcher (102) to describe the formation of extra-lymphoid sites, where memory lymphocytes and/or precursors can be re-stimulated by antigen to induce further clonal expansion or terminal effector responses. By definition, TLS arise in tissues whose main function is other than the generation of immune cells or the initiation of an adaptive immune response. This excludes the bone marrow and thymus, (as primary lymphoid organs) and spleen, lymph nodes and Peyer's patches (which are defined as SLOs). The kidneys, heart, pancreas, synovium and salivary glands are not embryologically predisposed to host the presence of lymphoid tissue therefore lymphocyte assembly at these sites should be considered TLS. The liver provides a hematopoietic function during embryonic development (103) however, this function is lost postnatally, thus including this organ among those that host TLS in adult life (97).

TLS form in response to a series of pro-inflammatory cytokines and TNF receptor family members following the local cross-talk between inflammatory immune cells and resident stromal cells. Fibroblasts, perivascular myo-fibroblasts and resident mesenchymal cells have been differently implicated in TLS development (39, 75, 104–112). Their role has been recently reviewed elsewhere (97, 113, 114). Probably evolved before SLO, TLS might have developed in ectopic tissues to fulfill the survival need of aggregated leucocytes, prior to placentation and development of SLOs. As such, the ability of TLS to be initiated independently from Lymphotoxin (LT) upon the expression of inflammatory cytokines and in absence of lymphoid tissue inducer cells (LTi) might have remained as heritage of their developmental ancestry (97).

Physiologically, the generation of a humoral response requires the physical interaction of naïve B cells with antigen experienced T cells within the confined space of a microenvironment rich in survival and chemotactic factors (115). Lymphocytes are recruited from the bloodstream to the SLO in response to a chemotactic gradient that regulates cell positioning and interactions (116, 117). CXCL13 and CCL19/CCL21, ligands for the chemokine receptors CXCR5 and CCR7, respectively, regulate the recirculation of naïve B cells between the inner part of the B cell follicle to the outer area of the T/B cell boundary (118), thus enabling the contact of B cells with antigen-experienced, activated T cells (119). Within the follicles, antigen-experienced B cells migrate toward the dark zone of the GC, a highly hypoxic CXCL12-rich area. Within this area they become highly proliferating centroblasts and upregulate the enzyme activation-induced cytidine deaminase (AID) (120, 121), that regulates the introduction of single base-pair substitutions of antibody gene segments in the immunoglobulin (Ig) variable-region genes, in a process defined as somatic hypermutation (SHM) (122).

**Figure 1:**
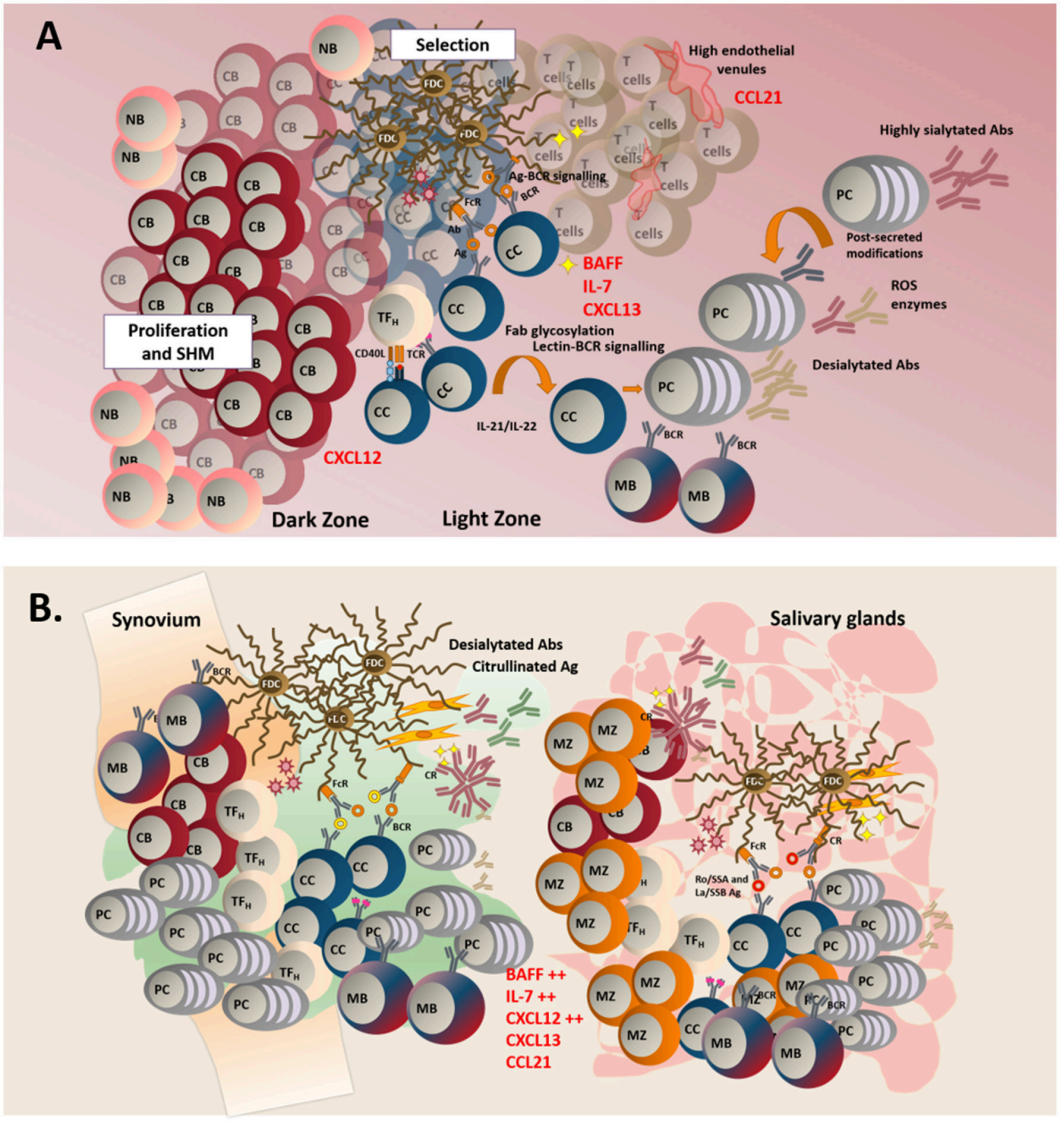
**(A)** In TLS, naïve B cells (NB), enter the follicle to initiate a classical germinal center reaction. In the dark zone, centroblasts (CB) proliferate and acquire somatic hypermutations in their variable region. In the light zone, centrocytes (CC) are selected after their interaction with specific antigen found on the surface of follicular dendritic cells (FDCs). Lectin-BCR signaling can potentially result in enhanced selection of B cells. Failure to receive survival signals from either TF_H_ (T follicular helper cells) or FDCs leads to B cell apoptosis. Successful affinity maturation results in either mature B cell (MB) of plasma cells generation. GC microenvironment can control the outcome of the immune response by regulating the glycosylation profile of the antibodies. **(B)** TLS display a less organized anatomical structure and a predominant infiltration of MB and marginal zone B cells (MB). Aberrant production of survival and chemoattractant signals is observed at these sites.

Following SHM, B cells stop proliferating and undergo the process of affinity maturation (123). Differentiated, non-dividing B cells (centrocytes) upregulate CXCR5 and migrate along the CXCL13 gradient toward the GC light zone (120), herby establishing connections with the network of follicular dendritic cells (FDC) that provide survival factors (124, 125) and antigen presentation via the CR2 receptor (125, 126). Within the light zone, centrocytes also encounter mature T follicular helper cells (T_fh_), known to provide signals for selection and terminal differentiation into long-lived plasma cells or memory B cells (127–130). Once exited from the GC, affinity matured B cells undergo the process of class switch recombination (CSR), that regulates isotype switching and ultimate effector function of the immunoglobulins (Igs). This latter process is also regulated by AID (130–142) (Figure 1A).

This organizational program in SLOs is maintained by the anatomical differentiation of specialized, resident stromal cells that regulate migration and functional activation of the immune cells in the different part of the follicle (138, 143–149). The development of this stromal network and the signals required for his homeostasis have been reviewed elsewhere (150). TLS display a similar anatomical structure to support naïve B and T cell recirculation, including the expression of homeostatic lymphoid chemokines CXCL13, CCL21 and CCL19 and the molecular complex peripheral node addressin (PNAd) (97, 98, 151, 152). However, the complex anatomical compartmentalization displayed in SLO is rarely acquired in TLS. While the majority of reports on TLS describe a certain degree of T/B cell segregation, vascular/stromal cell specialization and expression of lymphoid chemokines, the presence of a more complex organization of the TLS and the formation of functional GC is highly variable within and amongst diseases (4, 153–155). In TLS that form during chronic autoimmune processes, the establishment of such disorganized microenvironment, rich in survival factors and pro-inflammatory cytokines, but likely missing key checkpoints for autoreactive cells screening, is likely responsible for the local generation of pathogenic autoantibody specificities and oncogenic mutations, ultimately favoring disease progression (1, 9, 97, 98).

## TLS in autoimmune conditions: a lesson from rheumatoid arthritis and sjögren's syndrome

In 1996, Nancy Ruddle described the presence of a “structural chronic inflammatory process” caused by ectopic production of lymphotoxin, in the context of chronic inflammation of the pancreas (156). Since then, TLS formation has been associated with a localized process of inflammation at sites of infection, autoimmunity, cancer, and allograft rejection. The ultimate pathogenic role of TLS is still debated (98, 151) and most likely depends on the context, organ and type of disease. For the scope of this review we will focus on the role of TLS in supporting the autoimmune process in chronic autoimmune conditions and we will discuss the role of TLS in Rheumatoid Arthritis (RA) and primary Sjögren's Syndrome (pSS) (33, 36, 135, 151, 157–162).

RA is the most common rheumatic autoimmune condition, affecting 0.5–1% of the global adult population. The pathological features of the disease include severe inflammation of the synovial membrane that, in some cases, leads to tissue destruction and subchondral bone erosions (163–166). Histologically, the disease can be classified in 3 main histopathological subtypes: a lymphoid type, mainly characterized by T and B cell aggregates that form TLS; a myeloid type, characterized by diffuse infiltration of prevalent monocyte and macrophages; and the fibroid type, defined by scarce or no immune cell infiltration and prevalent synovial fibroblast hyperplasia (151).

The presence of a “…marked infiltration of chronic inflammatory cells *(lymphocytes or plasma cells predominating) with tendency to form “lymphoid nodules”* was recognized already in the 1957 RA classification criteria (167). In 1972, Munthe and Natvig suggested that the RA synovial membrane is similar to an active lymphoid organ, *containing many lymphoid follicles with GC that undergo local division and differentiation into plasma cells with restricted Ig production* (168). Later, Steere and colleagues described “*elements found in normal organized lymphoid tissue*” in synovial lesions from both RA and Lyme disease patients (169); suggesting that the formation of GC-like structures in the synovium is not specific for RA and can be driven by the local antigenic stimulation. It took, however, more than 40 years after these first descriptions to introduce the concept that B-cell affinity maturation could arise within the inflamed synovium (170). It is now accepted that TLS are present in less than half of RA patients who display so called “lymphoid” synovitis (151) and that, in those patients, the presence of TLS is associated with differential prognosis and disease manifestations (151). TLS formation in the synovia have been also identified in patients with psoriatic arthritis (171) and ankylosing spondylitis (172, 173).

A similar phenomenon of leucocyte aggregation in lymphoid like structures occurs in the salivary glands of patients affected by pSS, a disease characterized by chronic inflammation of the exocrine glands, with progressive loss of function (sicca syndrome) and systemic activation of the humoral response (174). Excessive B cell hyperactivity and extra-glandular manifestation are observed in ~30% of pSS patients and an increased risk for lymphoma development has been described in this condition. In 1974, Chused et al. first described the presence of lymphoid-like structures in the salivary glands of patients with pSS (175). This was followed by the report of local antigenic stimulation within GC-like structures in the salivary glands (176) and, 10 years later, by the description of FDC network formation within the aggregates (177). In 1998, Stott and colleagues provided the first experimental evidence of an antigen-driven GC response, defined by clonal B cell proliferation and clone hypermutation within the salivary gland inflammatory foci (37), and, since then, several features associated with lymphoneogenesis have been reported within pSS aggregates (157, 178).

It is now recognized that during pSS, TLS form in the minor salivary and/or parotid gland in around 30–40% of patients (151) and those structures host a phenomenon of oligoclonal B cell expansion and SHM of the Ig variable genes (37). The formation of TLS in pSS salivary glands correlates with increased B cell hyperactivity, the presence of anti-SSA and anti-SSB autoantibodies, hypergammaglobulinemia and cryoglobulinemia, supporting the hypothesis that TLS persistence contributes to disease progression in pSS (179). Our group has contributed to these reports, describing both the expression of lymphoid chemokines and of AID within highly organized aggregates that harbor in the salivary gland of patients with pSS and MALT lymphoma (135, 180). The relationship between TLS formation and disease progression in pSS is still debated. TLS detection has been associated with high antibody titer, systemic manifestations and lymphoma development. However, the direct correlation between GC formation in the salivary glands and lymphoma formation has not been demonstrated, suggesting that the development of GC+ TLS within the salivary glands represent one of the stages in the process of lymphomagenesis but is not *per se* sufficient to induce lymphoma (135, 154, 161, 180–182).

In order to better understand the pathogenic effect that TLS play in disease it is important to dissect the elements, present within these structures that contribute to their function and persistence in the tissue.

## Structural elements of TLS

### Antigen

There is enough evidence to support the hypothesis that TLS form to provide an immune response against locally displayed antigens. There are suggestions that TLS formation is an antigen (Ag)-driven process. In the mucosal associated lymphoid tissue that forms during Helicobacter *Pilori* gastritis antigen clearance following antibiotic treatment impacts on TLS maintenance and progression to lymphoma (183), similarly inducible bronchial associated lymphoid tissue can dissolve upon antigen clearance (184). Maffia and colleagues explored the properties of Ag presentation within TLS (58, 185) demonstrating that Ag presentation is regulated by a random process of diffusion, rather than selective Ag uptake by DCs. Those data are reinforced by the anatomical structure of TLS where conduits, able to support Ag movement and APC migration have been described (186). In this context, the absence of a capsule could favor not only the initial Ag delivery in the tissue, but the progressive accumulation of new antigen specificities during the course of the immune response, favoring the persistence of these structures in the tissue.

During a classical immune response, the antigens are collected by antigen presenting cells in the periphery and moved, via a complex network of lymphatic vessels, to draining lymph nodes (LNs) (187–189). LN space is pre-formed during the embryonic development and anatomically set before the generation of the immune response to accommodate optimal interaction between APC, Ag and immune cells. Differently by SLOs, TLS organization is not anatomically predisposed to organize such a response and Ag presentation is often provided by non-immune cells, such as stromal cells and epithelial cells (190–193).

Lack of Ag drainage could mechanistically explain TLS formation. TLS form spontaneously in the lungs of mice deficient for CCR7, a chemokine receptor required for the migration of antigen-charged dendritic cells (DCs) to draining lymph nodes (194). However, the reconstitution of these animals with CCR7-sufficient cells is enough to re-establish the physiological delivery of the antigen to the lymph node and to induce TLS resolution in the tissue. This evidence appears to suggest that an intrinsic defect in DCs is sufficient to trigger TLS establishment. However, it is not clear whether this phenomenon could be also supported by a defect of lymphatic drainage from the inflamed tissue.

The expansion of a functional network of lymphatic vessels is required for appropriate antigen delivery to the SLOs. There are several reports describing the dramatic remodeling of the lymphatic vessels during inflammation, whereby the activation of NF-κB pathway supported by the expression of LT, IL-1 and TNFα, stimulates the expression of Prox1 and increases the transcripts for the VEGF-R3, both of which are factors involved in lymphoangiogenesis (195–201). TLS lack the presence of an organized lymphatic system such as the one described in SLOs (152). However, the expansion of the lymphatic vascular system has been observed in these structures, in response to the same cytokine milieu that regulates the maturation of the non-vascular stroma at these sites (97, 105). It is not clear whether these newly formed vascular structures are, however, able to establish viable connections with pre-existing lymphatics. The failure to do so would prevent efficient drainage of the antigen to the SLOs and support the excessive antigenic stimulation in the peripheral tissue (89, 202–206).

Lymphangiogenesis associated with tertiary lymphoid structure (TLS) has been reported in numerous studies. Defects in lymphangiogenesis in RA present with a reduction in lymphatic flow, absence of lymphatic pulse and collapse of draining LNs is observed during disease and is associated with flare onsets as has been shown *in vivo* and *ex vivo* studies performed by Schwarz and colleagues (207). Accordingly, effective therapeutic approaches in RA, including anti-TNF and B cell have been associated with the expansion of the lymphatic bed (208) and increase in cell drainage from the synovium (209).

In a model of pSS our group demonstrated that during TLS assembly an expansion of the lymphatic vascular network takes place and this is regulated by the sequential engagement of IL-7 and LTβR signaling; suggesting the presence of a natural pro-resolving mechanism for lymphocyte exit from the tissues during TLS establishment (105).

The open questions related to the mobilization of Ag loaded APC to the draining SLOs could be addressed in the future by inducing TLS formation and tracking the movement of labeled antigen-loaded DCs across vessels. The possibility to interfere pharmacologically or genetically with the process of lymphoangiogenesis and with the molecules responsible for cell migration across these structures, is likely to elucidate this complex phenomenon and to provide evidences on the role of aberrant antigen presentation and vascular disturbances in TLS establishment and persistence.

Both RA and pSS are characterized by antibody production against a discrete set of autoantigens and a large body of research in this area has been focused around the identification of antigen specificities within the TLS in the context of these diseases. The presence of citrullinated proteins has been reported within the synovia of RA patients by Baeten (210) and others, and associated with the local expression of the enzyme peptidyl arginine deiminase (PAD) in patients characterized by high systemic and local levels of anti-citrullinated antibodies (APCA) (211). This report fails to demonstrate the presence of the citrullinated proteins within the synovial TLS and is in disagreement with other studies reporting the detection of citrullinated proteins in non-RA synovium lacking classical TLS (212); casting doubts on the exclusive association between citrullinated protein expression and TLS development in RA. Additional evidence that associate the presence of TLS with the generation of auto-antibody specificity against citrullinated peptides (but not necessarily local display of the defined antigen) will be discussed in a different section of this review.

Stronger evidences supporting the link between TLS and local auto-antigen presentation have been provided in pSS. Ro/SSA 52 kDa, Ro/SSA 60 kDa and La/SSB belong to a intracellular complex of RNA binding proteins that is physiologically involved in the intrinsic response to viruses (213). The aberrant expression of Ro and La has been reported in pSS patients upon cellular apoptosis or extracellular transport in vesicles (214–216). Moreover, the presence of anti-Ro52/TRIM21 specific plasma cells has been demonstrated, at the boundaries of well-organized TLS in pSS salivary glands, establishing a clear connection between local antigen presentation and TLS formation in this disease (158). The presence of extractable nuclear antigen (ENA) antibodies against these two ribonucleoproteins is pathognomonic for Sjogren's and associated with more severe systemic manifestation and worst prognosis (214, 216, 217).

### Lymphocytic components of TLS

We have recently reviewed the role of non-haematopoietic cells in TLS establishment and organization (97, 98) and for the scope of this issue focused on autoantibodies, we will limit the discussion in this manuscript to the lymphocytic compartment.

Whilst mainly constituted of B cells and associated with aberrant humoral responses and GC formation, TLS establishment and maintenance strongly relies on T cells. In humans, the presence of a shared TCR specificity among different follicles in the RA synovium, has been described, suggesting the presence of a common antigen for different TLS that form within the synovial tissue (218). In line with this finding, depletion of CD8+ T cells in human synovium-SCID mouse chimeras hinders the formation on TLS (218).

Recently, efforts have been made to identify the cells and signals required for TLS establishment and a series of reports have highlighted the important role of IL-17+ T cells. Th17 cells are required for iBALT formation (219) and for TLS establishment in a model of experimental autoimmune encephalomyelitis (EAE); the latter, dependent on the production of LT-αβ, IL-17 and IL-22 (22, 23, 220, 221). In human renal allograft rejection, Th17 cells have been shown to promote ectopic GC formation in an IL-21 dependent manner (222). Aberrant differentiation of Th17 cells in the absence of IL-27 has been also associated with aberrant TLS formation in an experimental model of arthritis and in a model of pSS (223, 224).

Our group has recently demonstrated the requirement of IL-22 producing T cells in the early phases of TLS establishment in murine salivary glands (39). In this model, IL-22 production, similarly to the IL-17 production in the lungs and brain, appears to regulate, independently but also in synergy with lymphotoxin and TNF, the ectopic production of lymphoid chemokines that defines TLS formation (97). These studies demonstrate that T cells, and in particular Th17/Th22 cells, play an important role in shaping the constituents of TLS in a manner that can support subsequent B cell recruitment and germinal center formation.

Whether TLS provide a site of aggregation for naïve T cell is not clear and whilst naïve T cell recruitment and priming has been reported within TLS that form in pancreatic tissue in NOD mice (225), it is more likely that effector T cells and central memory recirculate in these structures, in particular in the earliest phases of TLS assembly. On the contrary it is now well accepted that TLS function as a site for functional T cell polarization. TLS maintenance appears to hinge on the functional relationship between T-follicular helper cells and regulatory T cell populations. T cells displaying a T_fh_ phenotype have been described in TLS, where they are expected to regulate the GC reaction and the activation of resident proliferating B cells (1). In the TLS that form outside the arterial wall and control the atherosclerotic plaque development, the presence of T_fh_ correlates with the organization and maintenance of the ectopic B cell clusters (226). Functional interference of the T_fh_ by ICOS-L blockade results in decreased TLS formation and aberrant atherosclerotic plaque formation. The opposite effect is obtained by depletion of T regulatory cells, previously demonstrated to play a critical role in the homeostatic control of the TLS and in the atherosclerotic process (58).

The developmental program of T_fh_ in TLS is debated. There are suggestions that this population in TLS derives from a population of peripheral CXCR5^+^ T cells that migrate to the peripheral tissue following the newly established CXCL13 gradient. These circulating CXCR5+ cells do not bear classical signs of activation and would, by definition, preferentially migrate to SLOs; however, the local differentiation of HEVs and the expression of PNAd (the ligand for L-selectin) supports their homing to the TLS (227). Others suggest that, within TLS, T_fh_ locally differentiate from other T cell subpopulations, including Th17. In support of this hypothesis, in EAE, Th17 cells appear to acquire some characteristics of T_fh_ including the expression of CXCR5, ICOS and Bcl6 (23). Similarly, within the inflamed joints of RA patients, a population of PD1^hi^CXCR5^−^CD4^+^ T cells termed “peripheral helpers” has been described that appear to fulfill the function of T_fh_ within the periphery (228). In pSS the expansion of T_fh_ cells has been reported and correlates with the increasing frequency of memory B and plasma cells in the tissue and blood (229, 230).

Genetic manipulation in conditional knockouts is currently in use to induce TLS formation in mice deficient for specific T cell populations and will allow better definition of cellular requirements for TLS formation.

Classically, fully established TLS are mainly characterized by B cell infiltration and the inversion of the B/T cell ratio within TLS has been used as an index of disease severity (231, 232). In SLO, naïve B cells are known to receive antigen education and co-stimulation; however, whether a similar phenomenon would regularly occur in TLS is debated. Patients with pSS display altered peripheral blood B cell frequencies with a predominance of CD27^−^ naïve B cells, diminished frequencies/absolute numbers of CD27^+^ memory B cells in the periphery, and an enrichment of mature B cells in the salivary glands (233, 234). The presence of CD20^+^CD27^+^ B cells and plasmablasts is a consistent finding in pSS salivary glands biopsies (235). Whilst we have reported the presence of IgD^+^ naïve B cells, in particular in large TLS (180), memory B cells remain the predominant component of the infiltrates (180, 236). This casts doubts over the possibility that naïve B cells are primed within the TLS (235). In support of this hypothesis, *bona fide* GC B cells (CD10^+^CD21^+/−^CD24^+/−^CD27^−^CD38^+^IgD^−^ that express AID) are rarely found within the B cell aggregates of TLS, that are mainly inhabited by CD10^−^CD21^+^CD24^+^CD27^±^CD38^−^IgD^+^ marginal zone-like type II transitional B cells (159) (Figure 1B).

The connection between the marginal zone (MZ) and TLS establishment is also not clear. There are several evidences supporting the involvement of MZ B cells in autoimmunity, including reports of preferential SHM and B cell proliferation in MRL Fas/lpr mice spleen (237) and the presence of RF^+^ cells in the splenic marginal sinus bridging channels (238). The low threshold of BCR activation, the numerous effector functions of MZ B cells and the link between autoimmunity, TLS and MZ lymphoma development in pSS suggests a direct involvement of this population in TLS pathology (239, 240). However, the origin of the MZ-like B cells and the relationship between those and the ectopic GC has not been proven. In humans, MZ B cells are allegedly able to recirculate and carry a highly mutated B cell receptor (241–243), thus suggesting a post-GC origin of this population. This is not the case in mice, where MZ B cells are stable and permanently located in the spleen (242–244). Interestingly, however, MZ-B cells in humans share some phenotypic features of transitional B cells, a highly autoreactive B cell population that emerge from the BM and mature inside the spleen before entering the follicle (245–248), suggesting the possibility that transitional immature autoreactive cells are inhabiting the ectopic follicles. The recirculation pattern and screening of transitional B cells has been described from Spencer and co-authors in an elegant work that describes the migration and BCR editing of this population in the gut-associated lymphoid tissue (GALT) (245). This process, aimed at modifying the specificity of autoreactive clones, is altered in systemic lupus erythematosus (SLE), resulting in the expansion of the autoreactive B cell repertoire (245). In diseases characterized by TLS formation, such as pSS and RA, this recirculation pathway could be also altered, favoring the migration of autoreactive clones from the lymphoid organs to the TLS. Hereby, the aberrant expression of survival factors and chemokines would support clonal expansion in the absence of BCR editing and support persistence of autoimmunity.

The use of mass cytometry on digested tissue and sections are needed to better characterize in humans the phenotype and functional features of the B cells inhabiting the TLS. The use of transgenic mice engineered to track cells *in vivo* (249) will be useful in inducible models of TLS to perform migration studies *in vivo*.

## TLS as aberrant microenvironments for autoreactive B cell survival and differentiation

More than simply acting as a hub for lymphocyte migration, TLS have also been shown to provide critical survival signals for incoming lymphocytes and differentiated long-lived plasma cells such as BAFF, IL-7, and CXCL12 (98, 250). The persistence of TLS in the tissue, despite peripheral B cell depletion of post Rituximab, has been reported in RA (251), SS and lymphoma (252) and, more recently in peri-bronchial TLS described in two patients with cystic fibrosis and chronic Pseudomonas aeruginosa infection treated with B cell depletion therapy before transplantation. The reason for this persistence most probably resides on the excess survival factors, such as B cell activating factor (BAFF) or IL-7 present within the TLS that protects tissue infiltrating cells.

BAFF is a potent B-cell survival factor produced within SLO GCs and in the periphery by fibroblasts and epithelial cells (159, 248–253) Excess BAFF is known to rescue self-reactive B-cells from peripheral deletion and allows their entry into forbidden follicular and marginal zone niches (253). The connection between BAFF, MZ B cells, loss of tolerance and TLS emerged from studies in mice transgenic for BAFF (BAFF-Tg), that develop a lupus-like syndrome followed by infiltration of MZ-like B cells within salivary glands TLS (254). Interestingly, BAFF-Tg asplenic mice that lack MZ-B and B1a cells, but retain normal B1b cell numbers, develop lupus nephritis but lack TLS in the salivary glands, suggesting that both BAFF and MZ-B cells are required for TLS establishment in this model (255).

Other lymphoid survival cytokines including IL-7 have been described in association with TLS establishment in chronic diseases (162, 256–258). Gene expression levels of IL-7, IL-7 receptor (both IL-7Rα and IL-2Rγ subunits) and its downstream signaling gene JAK3 are significantly elevated in RA patient biopsies displaying TLS (259). Similarly, engagement of the IL-7/IL-7R axis has been linked to formation of TLS in salivary glands and associated with pSS pathology (22, 23, 33, 36, 37, 39, 58, 75, 89, 102–112, 112–262). Among other critical homeostatic functions, IL-7 can abrogate the suppressive ability of Treg, altering the balance between pro-inflammatory effector cells vs. suppressive T cells (162, 256, 258). Consistent with these observations, *in vivo* studies demonstrated the ability of IL-7 to induce TLS formation (263–265). The reciprocal expression of T and B cell survival factors in TLS is somehow strictly regulated by the critical balance between infiltrating T and B cells, probably in response to gradients of lymphotoxin and TNF family members. The mechanism regulating this production and the resulting segregation of lymphocytes in T or B cell rich areas is still under investigation (266).

We and others have provided evidence that a functional GC response takes place within these structures. This supports the concept that even if TLS do not initiate disease they are involved in its progression. In particular, we have demonstrated that AID is expressed in pSS salivary gland TLS in association with networks of follicular dendritic cells (135) and that its expression is retained in the large GCs found in parotid pSS-MALT lymphomas. On the contrary, neoplastic B cells are found to be consistently negative for AID expression (135). AID expression in GC B cells controls susceptibility to apoptosis, ultimately regulating the magnitude of the GC response (267). In SLO, low levels of AID expression have been associated with defective somatic hypermutation and decreased peripheral B cell tolerance (268). AID expression in TLS is consistently low (as compared to SLOs), thus potentially explaining the aberrant survival and lack of selection of autoreactive B-cell clones in ectopic GCs.

Other data have been generated supporting the functional role of TLS in sustaining the generation of novel antibody specificities. Transplantation of TLS from pSS salivary glands infected with Epstein-Barr virus (EBV) into SCID mice have been shown to support the production of anti-Ro 52/anti-La 48 and anti-EBV antibodies and the survival of autoreactive B cell clones (158). Similar data have been produced for RA. The presence of CD138^+^ plasma cells, characterized by immune reactivity against citrullinated fibrinogen, has been described within AID^+^/CD21^+^ follicular structures (33). Moreover, the survival of these clones in a transfer model of human biopsies in SCID mice, alongside the detection of gamma-Cmu circular transcripts in synovial grafts, has been reported. These observations provide evidence that synovial TLS represents an independent compartment for B cell maturation (33).

## Autoantibody production goes local

The contribution of the immune response that arises within TLS toward disease severity, including the production of autoantibodies, remains controversial (151). Nonetheless there are substantial evidences in support of local antibody production within the inflamed synovium and convincing documentation that the synovial microenvironment could independently favor the production of RA specific antibodies (33)

Mellors et al. firstly described the presence of “plasma B cells” that are able to react with FITC-labeled human IgG, interpreting this result as evidence of synovial production of rheumatoid factors (RF) by tissue-resident plasma cells (269). The first solid indication of local IgG production in RA is dated to 1968 with the report of Ig synthesis in rheumatoid synovium *in vitro* (270). Further studies supported this observation suggesting that gene selection, usage of kappa/lambda chains and class switching follows a non-stochastic process in the RA synovium (168). Similarly, the enrichment in RF^+^ B cells producing mono-reactive, affinity matured, class switched antibodies in the RA synovium is highly suggestive of a local process of affinity maturation (271–273). On the contrary, clones producing mono-reactive RF have not been obtained from the synovial tissue of patients with osteoarthritis, where TLS do not form, supporting the link between chronic autoimmune diseases and TLS (271–275).

The production of anti-citrullinated protein antibodies (ACPA) has been firmly associated with RA development (276) and there are convincing evidences that these specificities can be locally produced in the RA synovium within the TLS (277, 278). Both anti-cyclic citrullinated peptide (CCP) antibodies (279) and anti-CCP producing B cells (280) have been detected in the synovial fluid of RA patients and antibodies against different citrullinated RA candidate antigens (vimentin, type II collagen, fibrinogen and α-enolase) appear to be enriched in the joint compared to paired serum (281). Notably, the presence of anti-CCP antibodies in the synovium has been also reported in RA seronegative patients (279, 282), thus highlighting the dissociation between the systemic and local autoimmune response. In support of this notion, the presence of FcRL4^+^ ACPA producing IgA-B has been reported in the synovium, but not in the blood of RA patients (283). This observation provides an indication that inflammatory joints provide a specific microenvironment able to shape and influence B cell immune phenotype and output.

The ability of TLS to sustain the whole autoimmune process in the absence of SLO is debated. However, cloning of the local B cell repertoire isolated from inflamed organs bearing TLS is highly suggestive of the presence of a functional and SLO-independent process of affinity maturation. Terminally differentiated CD20^−^CD38^+^ cells, rheumatoid factor (RF) producing B cells have been detected in the inflamed joints of RA patients (284). Moreover, clonal analysis has provided evidence of an antigen-dependent process of SHM, selection and isotype switching in TLS positive RA synovium, indicating that a dominant antigen-specific local immune response shapes the synovial plasma cell repertoire (170, 285–290). Similarly, in pSS, the multiclonal expansion of B cells within the salivary glands has been described. Expansion of B cell clones bearing Humkv325, a conserved V kappa gene usually associated with lymphomas, was described previously in 1989 (291). Additional studies further supported the notion that an antigen-driven germinal center-type B cell response and somatic hypermutation occurs within the salivary glands (37, 292, 293). The presence of clones that expand and mature in the TLS does not prove that the autoimmune process is initiated within the TLS, or that the presence of TLS is causative of disease. However, a certain degree of antigen-experience and affinity maturation of the B cell repertoire undoubtedly occurs within TLS (33, 135, 153, 160, 294–296) and, whilst the causal role of these structures in disease initiation cannot be proved, TLS certainly display the ability to host and perpetuate the autoimmune process. Production of Ig and RF has been shown in other tissues, in addition to the synovium, including rheumatoid pericardium (297), pleura (298), muscles (299), and in the inducible bronchus-associated lymphoid tissue (iBALT) in patients with pulmonary complications of RA (219). The presence of sputum autoantibodies in the absence of systemic seropositivity, and the increased autoantibody:total Ig ratio in the sputum (300) suggest that lymphoid tissue present in the bronchi of RA lungs can also act as sites of antibody development.

Independent IgG and IgM synthesis has been also described in pSS salivary glands (301) with later studies confirming the presence of RF^+^ clones in ~43% of patients with pSS (302) and with the ability of salivary gland infiltrating B cells to secrete antibodies specific for the Ro52/TRIM21, Ro60 and La autoantigens (36, 179, 217, 303). *In vitro* expression of recombinant antibodies derived from either newly emigrant/transitional mature naïve B cells from pSS patients and healthy individuals confirmed the presence of high frequencies of autoreactive antibodies in both populations. This suggests a general defective peripheral B cell tolerance in this condition (304).

Analysis of Ig levels in different compartments (blood, saliva) has further contributed to our understanding of the ability of TLS to independently produce antibodies. Increased levels of IgA, but not IgG- and IgM-RF, has been detected in the saliva of patients with pSS (305). A study on isotype distribution of anti-Ro/SS-A and anti-La/SS-B antibodies in the plasma and saliva of patients with pSS demonstrated a correlation between the focus score (the measured degree of salivary gland inflammation) and autoantibody titers in saliva or blood. This report establishes a pathogenic link between locally displayed autoantigens, presence of antigen specific B cells in the inflamed tissue and autoreactive Ig levels (306).

## Immunoglobulins and glycosylation: the sweeter the better?

It is becoming increasingly clear that antibody post-translational modifications, in particular glycosylation, can influence their function and pathogenicity. However, a relationship between the pathogenic microenvironment established in the TLS and the progressive acquisition of pathogenic post-translational modifications has not been demonstrated.

Glycosylation is the process by which glycans are attached to proteins, lipids and other molecules, thereby altering their structure and influencing their biological activity. Whilst IgG presents a single conserved N glycosylation site within the Fc region, other subclasses are more heavily glycosylated (307). IgG Fc glycosylation determines the binding of the globulins to their receptors, FcRs type I (FcgammaRs) and II (SIGN-R1, DC-SIGN, DCIR, CD22, and CD23), thereby influencing Ig downstream pro-inflammatory, anti-inflammatory or immunomodulatory effects (308, 309). In addition to conserved IgG Fc glycans, ~15–25% of serum IgG contain glycans within the Fab domain. Intriguingly, the attachment sites for N-glycans to the Fab portion is determined by the process of somatic hypermutation and, accordingly, Fab glycosylation could influence antibody binding, activity, half-life, formation of immunocomplexes and strength of BCR signaling [extensively reviewed in (310)].

The presence of altered glycosylation in RA was suggested in the 1970s, but it wasn't until 1985 when two studies from Oxford and Japan demonstrated different galactosylation profiles between normal individuals and patients with RA or OA (311). Later, Axford and colleagues reported the presence of reduced circulating B cell galactosyltransferase activity in RA (312), which was later confirmed in other studies (313–315). Other post-translational modifications have been described in RA. Several studies have demonstrated the presence of an altered overall glycosylation status within specific Ig subclasses (316) that can be detected before disease onset (317). This correlates with measures of disease activity (318, 319) and decreased sialylation of RF-IgG, but not in non-RF-IgG (318, 320, 321).

More recently, the degree of IgG glycosylation has been used to monitor treatment effectiveness (321) and, whilst no differences have been observed in the Fc glycosylation pattern between ACPA-IgG1 and total IgG1 in arthralgia patients, a decrease in galactose residues have been observed in patients with preclinical synovitis before the onset of RA; a change probably supported by the increasingly inflammatory microenvironment (322). The increased presence of agalactosylated IgG in the synovial fluid as compared to serum samples of RA has also been reported (323). Finally, Scherer et al. recently demonstrated the presence of autoreactive IgG in synovial fluid with decreased number of galactosylation and sialylation sites as compared to serum. This latter difference appeared to be specific for autoreactive specificities as no difference was observed in total IgG glycosylation (324).

Elevated levels of asialylated IgG have been detected in 60% of pSS patients and those appear to correlate with clinical manifestations, such as Raynaud's phenomenon and arthritis. A strong correlation with rheumatoid factor or IgA-containing immune complexes was reported (325). Based on IgG galactosylation, the pSS patients can be classified into two groups: one with comparable galactosylation status as in RA patients with the presence of RF, and the other similar to healthy individuals, and RF seronegative (326).

More recently, studies on Fab glycosylation and disease have been performed. Corsiero and colleagues reported the relationship between increased molecular weight of anti-NET antibodies and the presence of N-glycans onto the Fab domain of autoreactive clones in RA, suggesting that the process of SHM occurring in the synovium is responsible for the acquisition of-N glycosylation sites (286). Acquisition of N-glycosylation sites and subsequent enrichment in Fab-glycans in the variable domain of ACPA-IgG has been further confirmed (327, 328). On a similar note, it has been reported that there is a selective increase in Fab-N glycosylation sites in ACPA specific clones. However, the presence of those glycans didn't appear to significantly alter the antigen binding of the APCA. Accordingly, *in silico* analysis suggested that the added glycans were not located on the antibody binding sites (329). Moreover, an increased frequency of N-glycans in the Fab ACPA domain, but in association with altered antibody affinity, has been also reported (330). Interestingly, this enrichment was more prominent on ACPA isolated from synovial fluid compared with peripheral blood (264), providing evidence that the local microenvironment influences the immunoglobulin glycosylation pattern. Hamza *et al*. recently reported the high prevalence of acquired IgG N-glycosylation sites in pSS suggesting a hypothesis that in pSS, the selection pressures that shape the antibody repertoire in the parotid glands results from an antigen-independent mechanism and is driven by interactions between glycosylated B cell receptors and lectins within the microenvironment (328). In summary, the glycan composition can have different associations with the disease, depending on the site of glycosylation. Decreased and increased glycosylation for the Fc and Fab portion, respectively, have been associated with RA and SS.

A relationship between post-translational modifications and antibody pathogenicity has been proposed. Leader et al. reported the presence of agalactosylated IgG in synovial immunocomplexes, suggesting a pathogenic role for agalactosylated IgG (331). However, the relationship between glycosylation and RF activity is debated (318, 332). The presence of N-linked glycosylation sites within the Fc portion of target IgG has been also shown to markedly reduce RF binding *in vitro* (333) whilst the ability of rheumatoid factors to selectively bind hypogalactosylated IgG has been suggested (334). In mice, desialylated but not sialylated immune complexes appear to enhance osteoclastogenesis *in vitro* (335). Accordingly, artificial sialylation of anti-type II collagen antibodies, including ACPAs, but not other IgG can supress the development of collagen-induced arthritis (CIA) (320, 336).

A potential pathogenic role of IgG glycosylation in pSS pathogenesis, to our knowledge, has not been addressed yet. A recent study pointed out that the Fc-mucin binding is enhanced when antibodies are agalactosylated, offering a mechanistic concept for increased binding on mucosal surfaces of the inflammatory agalocysylated antibodies and potential antibody pathogenicity (337).

Agalactosylated IgG levels were not found to be correlated in twin pairs indicating a low influence from genetic factors for IgG glycosylation (338). However, four loci contained genes for glycosyltransferases (ST6GAL1, B4GALT1, FUT8, and MGAT3) have been highlighted in genome-wide association studies for loci associated with IgG N-glycosylation (339). There is evidence to support the notion that the microenvironment can influence Fc IgG glycosylation. A recent study illustrates the ability of CpG, IFN-gamma and IL-21 to increase Fc-linked galactosylation and reduce bisecting N-acetylglucosamine levels (340). Stimulation of a mouse B cell lymphoma line with IL-4 and IL-5, but not LPS, has been shown to significantly decrease the terminal glycosylation of secreted IgA (341) and IgM (342), but substantially increase the terminal glycosylation of MHC Class-I (342), suggesting that the glycosylation machinery works in a protein-specific manner.

A mechanistic link between inflammation and post-translational modification has been recently established by G. Schett's group in a manuscript illustrating the ability of IL-21 and IL-22 to regulate the expression of α2,6-sialyltransferase-1 in newly differentiating plasma cells, thus controlling the glycosylation profile of secreted IgG (320). Interestingly, both T cell-independent B cell activation (343) and tolerance induction with T cell-dependent protein antigens (344) results in the production of sialylated IgG. However, T cell independent vaccination seems to result in a stronger induction of sialylated antigen specific antibodies (345).

IgG glycosylation can also be controlled at an extracellular level. IgG sialylation has been reported in the bloodstream, through secreted ST6Gal1 (326). S-glycosyltransferases have also been shown to alter the IgG molecule at sites of inflammation with local platelets serving as nucleotide-sugar donors (346). Other reports link the process of altered glycosylation to a post-secretory degradative process involving oxygen free radicals (347). All together these reports suggest the possibility that the site of antibody synthesis can profoundly affect the post-translational profile of the immunoglobulins.

Due to technological limitations, the extent of the disease-related glycan alterations and the role of these modifications in disease pathophysiology has not been thoroughly addressed. A novel microfluidic-based method to identify trace sulphated IgG N-glycans as biomarkers for rheumatoid arthritis has been recently described (348) and high-throughput methods for analysing IgG glycosylation have also been introduced (349). These tools have been only used in selected populations and their application on a larger scale could, in the future, unveil differences and patterns not yet captured.

To our knowledge, there has been no attempt to use these stratification tools in the context of TLS associated pathologies. The differential profile of glycosylation observed in Ig isolated, respectively from serum and synovial compartments suggest the fascinating hypothesis that SLO and TLS differentially regulate these post-translational modifications (323, 324, 350). However, the possibility that Ig derived from SLO and TLS present substantially different “sugary fingerprints” and that those patterns correlate with a certain degree of tissue involvement and disease progression has still to be proven.

## TLS and lymphomagenesis

If the concept of an association between progressive post-translational modifications of the Ig repertoire and continuous antigen exposure within a highly inflammatory environment is true, we should be able to detect progressive accumulation of Ig in patients undergoing malignant transformation through the course of autoimmunity. The occurrence of non-Hodgkin's lymphoma (NHL) is pathogenically linked to TLS and represents the most serious complication of pSS, but not RA (351).

B cell VH and VL gene analysis for pSS patients with lymphoma revealed several point mutations in the germline genes and intra-clonal sequence heterogeneity, in line with an ongoing somatic hypermutation process sustaining lymphoma growth (352, 353). It is believed that the emergence of monoclonal RF B cells within the polyclonal infiltrate of the salivary gland TLS represents a key developmental step in lymphomagenesis. Chromosomal abnormalities and mutation eventually converge in these B clones that present a proliferative advantage, ultimately converting them into malignant clones. Indeed, there is a strong bias for RF specific B cells in the salivary gland MALT-type lymphoma (354–356), whilst alternative analysis of the B cell repertoire in micro dissected labial salivary glands could not convincingly demonstrate predominance of RF reactivity in the infiltrating clones (357). The relationship between GC formation and lymphomagenesis has been recently challenged and further studies will be required to clarify the pathogenic link between TLS persistence and emergency of malignant clones (161, 182, 358, 359).

The high incidence of acquired N-glycosylation sites found in follicular lymphoma (360) would be suggestive of a similar phenomenon in pSS associated MALT lymphoma; however, contrary to these expectations, patients with MALT lymphoma present low frequency of N-glycosylation sites (161, 182, 358, 359, 361). Longitudinal analysis of the glycosylation and sialylation profile in patients with TLS undergoing lymphoma transformations are needed to address these questions.

## Future prospective and conclusions

In conclusion, TLS formation can be easily considered as a hallmark of tissue autoimmunity. In the past few years a large body of work has been generated aimed at dissecting key aspects of TLS biology, however, many areas have to be further addressed. The inter-dependency between SLOs and TLS has to be better dissected in order to understand whether these immune hubs are functional, both in the early phases as tolerance is broken and, later, during disease progression. The signals regulating migration pathways and differentiation of immune cells within the TLS should also be investigated *in vivo* with the prospective to target these pathways therapeutically. A better knowledge around the signals involved in TLS establishment and maturation, but in particular, the mechanisms regulating GC formation and regulation should be acquired. Moreover, a specific effort should be made to dissect the functional role of TLS GCs in the development of lymphoma. Finally, key questions should be answered around the cross-talk between the TLS and their surrounding environment, dissecting the permissive factors for TLS formation and persistence in the tissue.

The acquired knowledge on the role of non-haematopoietic stromal cells in TLS biology has been critically important in explaining why these structures are resistant to classical immune cell therapy. In the future, potential combined therapy could be utilized to interfere with the microenvironment alongside targeting immune cells in TLS associated disease that is non-responsive to classical immunosuppression.

## Author contributions

EP and FB defined the content of the manuscript, contributed to literature search and manuscript writing. SN, DG and SC contributed to literature search and manuscript writing. CS contributed to manuscript writing. EP and FB created graphical illustrations. All authors approved the final version of the manuscript.

### Conflict of interest statement

The authors declare that the research was conducted in the absence of any commercial or financial relationships that could be construed as a potential conflict of interest.
